# Ferroptosis in veterinary medicine: mechanisms, therapies, and unmet challenges

**DOI:** 10.1080/01652176.2025.2569558

**Published:** 2025-10-08

**Authors:** Mohammed Zayed, Mohamed Abdelrazek, Byung-Hoon Jeong

**Affiliations:** a Korea Zoonosis Research Institute, Jeonbuk National University, Iksan, Republic of Korea; b Department of Bioactive Material Sciences, Jeonbuk National University, Jeonju, Republic of Korea; c Department of Surgery, College of Veterinary Medicine, South Valley University, Qena, Egypt; d Faculty of Veterinary Medicine, Cairo University, Giza, Egypt

**Keywords:** Ferroptosis, iron metabolism, reproductive diseases, nutritional diseases, veterinary medicine, therapeutic strategies

## Abstract

Ferroptosis, a regulated cell death, has emerged as a critical contributor to various pathological conditions in animals, particularly infectious, reproductive, musculoskeletal, and nutritional diseases. Ferroptosis differs from apoptosis, necrosis, and other types of cell death, being characterized by the production of reactive oxygen species and excessive lipid peroxidation. Research indicates a close interplay between oxidative stress and ferroptosis in veterinary contexts, where pathogens may manipulate ferroptosis to alter host immune responses, underlining its role in disease progression and defence mechanisms. Key regulators such as SLC7A11, ACSL4, and FSP1 have also been implicated in ferroptosis-related pathophysiology across animal species. Nutritional deficiencies, such as selenium deficiency, impair the activity of GPX4, a key antioxidant enzyme that suppresses ferroptotic pathways. Several therapeutic strategies, such as antioxidants, ferroptosis inhibitors, nutritional supplements, and iron chelators, are currently being explored in veterinary medicine, requiring tailored approaches due to metabolic differences among species. Despite increasing attention in human medicine, ferroptosis remains poorly understood in animal health. Therefore, this review consolidates current knowledge on ferroptosis in veterinary medicine and explores its mechanistic contribution to the pathogenesis of animal diseases. We highlight the emerging strategies for therapeutic intervention and improved animal health outcomes.

## 1. Introduction

Programmed cell death (PCD) is a fundamental biological process critical for tissue homeostasis, embryonic development, and disease regulation in animals (Fuchs and Steller 2011; Chen et al. 2024). By removing excess, damaged, or dysfunctional cells, PCD establishes proper tissue remodelling (Kuranaga et al. 2011; Chen et al. 2024) and regulates immune responses (Miura 2011). Dysregulation of PCD is implicated in various pathologies, including cancer-related diseases, neurodegenerative disorders, cardiovascular diseases, respiratory and renal diseases, digestive disorders, and developmental abnormalities (Fuchs and Steller 2011; Chen et al. 2024). Apoptosis, necrosis, autophagy, and pyroptosis are the most recognized forms of PCD (Green 2019; Shen et al. 2023). However, the recent discovery of ferroptosis—an iron-dependent form of PCD—has expanded our understanding of cell death mechanisms. Characterized by distinct biochemical, metabolic, and morphological features, ferroptosis is driven by iron-mediated oxidative damage to phospholipid membranes, often triggered by depletion of glutathione peroxidase 4 (GPX4) or disruptions in iron metabolism (Dixon et al. 2012; Dang et al. 2022; Gan et al. 2022). Recent studies have established a connection between ferroptosis and various conditions such as neurodegenerative disorders, cancers, ischemia-reperfusion injury, reproductive issues, and osteoarthritis (Dixon et al. 2012; Jiang et al. 2021; Liu et al. 2023a; Fei and Ding 2024; Zhou et al. 2024). Since its discovery in 2012, research across multiple species, including humans, rodents, livestock, and companion animals, has focused on unravelling the mechanisms underlying ferroptosis, offering insights into its therapeutic potential in human and veterinary medicine. Different strategies and reagents have been developed to target ferroptosis, such as antioxidants, phytochemicals, and cell-based therapies (Zhao et al. 2022; Cui M et al. 2024; Jiao et al. 2024; Zayed et al. 2025). Nanobiotechnology has also emerged as a promising tool to enhance the therapeutic efficacy in ferroptosis-associated diseases (Han et al. 2024).

**Figure 1.**
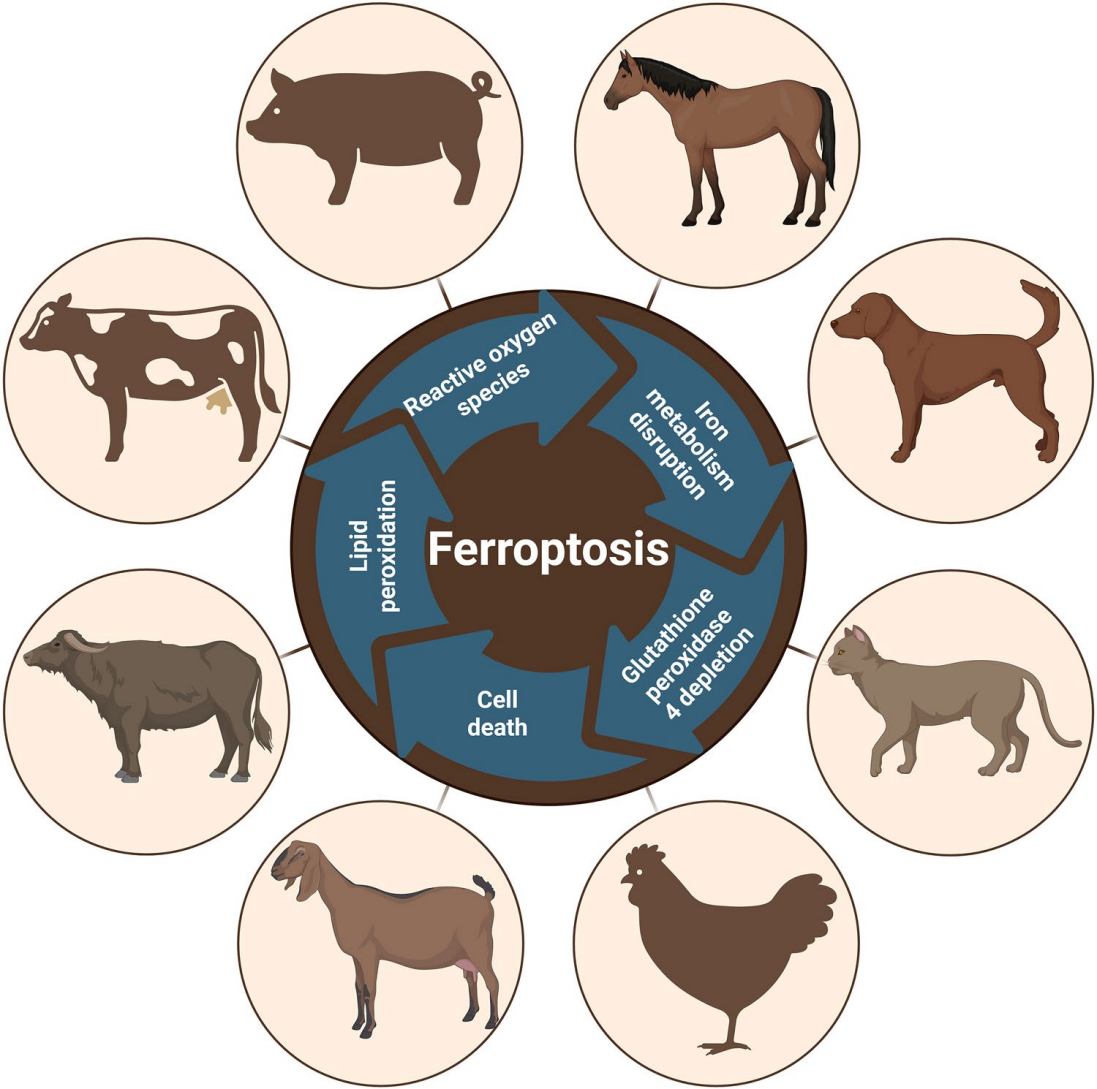
Schematic representation showing the involvement of ferroptosis among animal species. Created with https://www.biorender.com.

In veterinary medicine, ferroptosis has emerged as a critical mechanism for understanding the pathogenesis of various animal diseases and developing targeted therapeutic strategies (Figure 1). Companion animals and livestock are particularly susceptible to conditions impaired by oxidative stress, including metabolic syndromes, inflammatory diseases, and aging-related disorders (Blanca et al. 2024). Despite increasing research interest, the role of ferroptosis in veterinary medicine remains insufficiently investigated. This review discusses the current knowledge on ferroptosis mechanisms in animal diseases. Furthermore, we highlight how modulating ferroptosis may improve both diagnostic accuracy and therapeutic outcomes in animal health, bridging the gap between experimental research and clinical applications.

## 2. Search strategy

We conducted a thorough search in major scientific databases, including PubMed, Scopus, and Web of Science, for original research and review articles published in English. Articles on ferroptosis mechanisms and therapeutic approaches in domestic animals and poultry were included. Additionally, articles discussing nutrition and food additives targeting ferroptosis were also considered. The search terms used were: ferroptosis, iron-dependent cell death, lipid peroxidation, veterinary, animals, livestock, pigs, poultry, and nutrition. We combined the terms: (ferroptosis or GPX4) and (cattle, pigs, or canine). Finally, we screened the reference lists of the retrieved articles to identify further relevant studies. No publication date restrictions were applied.

## 3. Pathways of ferroptosis

The pathways of ferroptosis have been extensively reviewed (Gaschler and Stockwell 2017; Stockwell et al. 2017; Tang et al. 2021; Stockwell 2022; Zayed et al. 2025). Ferroptosis can occur through several major pathways related to iron and lipid metabolism and the glutathione-GPX4 axis. This section summarizes the current fundamental molecular mechanisms and regulatory pathways of ferroptosis.

### 3.1. Iron metabolism pathway

**Figure 2.**
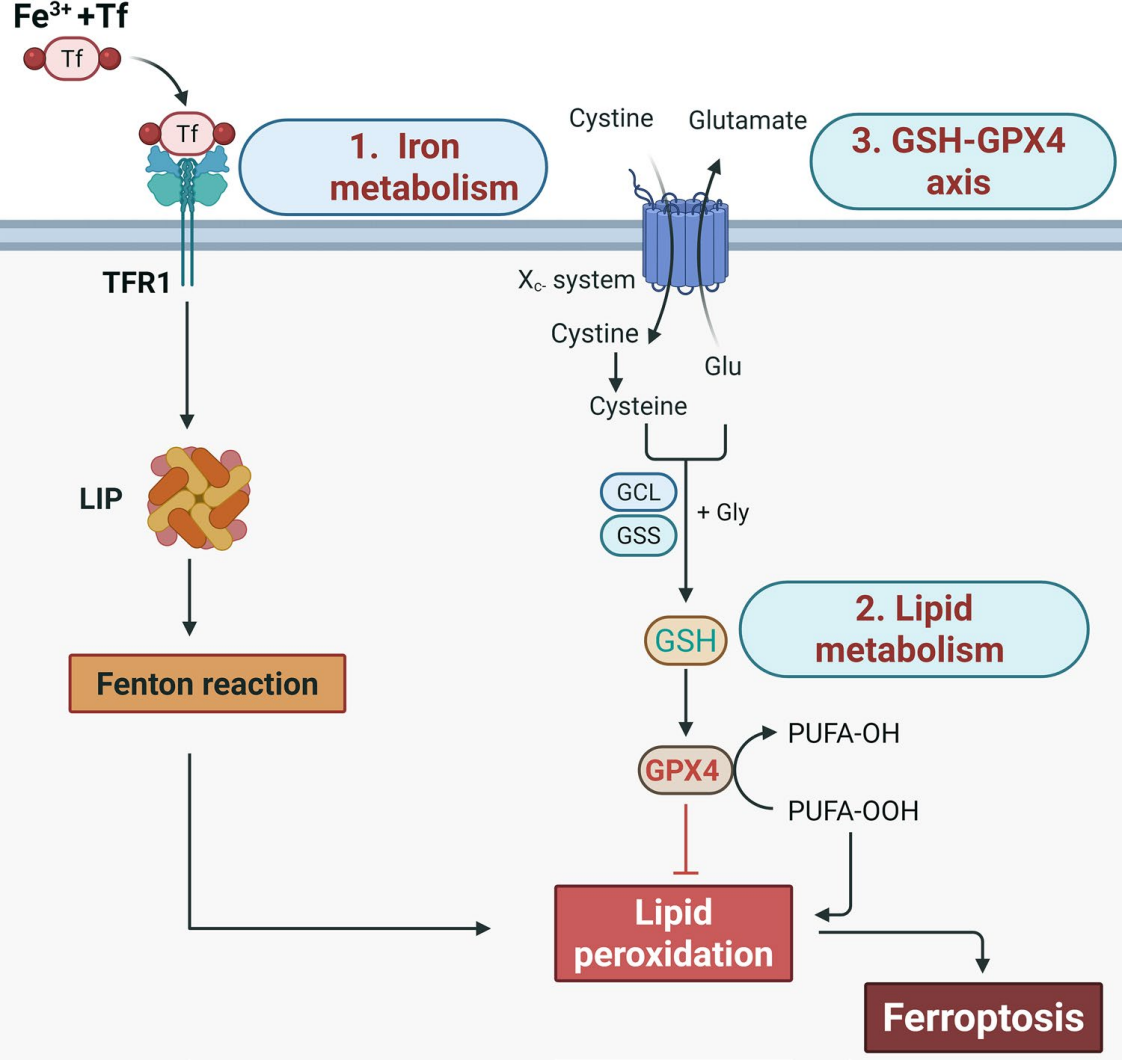
Mechanisms of ferroptosis. The core metabolic drivers of ferroptosis include iron metabolism dysregulation, lipid peroxidation, and failure of the GSH/GPX4 defence system. Cellular iron is primarily acquired through transferrin-bound iron uptake *via* TFR1-mediated endocytosis. Liberated Fe^2+^ ions fuel the generation of lipid peroxides *via* Fenton chemistry. The system Xc^-^ antiporter imports extracellular cystine, exchanging it for intracellular glutamate. Once inside the cell, cystine is reduced to cysteine, which serves as the essential substrate for synthesizing GSH through the sequential actions of GCL and GSS. GPX4 critically depends on GSH to reduce cytotoxic lipid hydroperoxides to non-toxic lipid alcohols. GSH, glutathione; TFR1, transferrin receptor-1; LIP, labile iron pool; GPX4, glutathione peroxidase 4; GCL, glutamate-cysteine ligase; GSS, glutathione synthetase. Created with https://www.biorender.com.

Iron metabolism plays a central role in regulating ferroptosis (Figure 2). Labile iron, particularly Fe^2+^, acts as a catalyst for reactive oxygen species (ROS) generation *via* the Fenton reaction, thereby promoting lipid peroxidation in cellular membranes (Dixon et al. 2012). Cellular susceptibility to ferroptosis depends on the balance between iron uptake, storage, and export. Dysregulation of this balance, such as iron overload or deficiency, sharply alters susceptibility to ferroptotic cell death (Stockwell et al. 2017; Miao et al. 2024). Iron is maintained in a dynamic balance through cellular and molecular regulatory mechanisms (Yang and Stockwell 2016; Catapano et al. 2025). Extracellular Fe³^+^ binds to transferrin (Tf) and is internalised *via* transferrin receptor 1 (TFR1) (Figure 2). Within acidic endosomes, Fe³^+^ is reduced to Fe^2+^ by six-transmembrane epithelial antigen of the prostate 3 (STEAP3) (Aisen 2004; Gao et al. 2015). Divalent metal transporter 1 (DMT1) then transports Fe^2+^ into the cytosol, where excess iron is stored in ferritin, a heteropolymer composed of ferritin light chain and ferritin heavy chain 1 (FTH1) (Gunshin et al. 1997; Ohgami et al. 2005). During periods of iron deficiency, ferritin undergoes selective autophagy (ferritinophagy), mediated by nuclear receptor coactivator 4 (NCOA4), to release stored iron into the labile pool (Dowdle et al. 2014). Mitochondria are key consumers of intracellular iron, utilising it for heme biosynthesis and iron-sulphur cluster assembly, both of which are critical for electron transport chain function, redox reactions, and DNA repair (Xu et al. 2013; Gao et al. 2019). Iron-responsive element-binding proteins (IREBs), including IREB1 and IREB2 (IRP2), further regulate iron metabolism by binding to iron-responsive elements in mRNAs. IREB1 is highly active under high oxygen conditions, while IREB2 regulates cytoplasmic iron levels and is active under low oxygen conditions (Meyron-Holtz et al. 2004; Wilkinson and Pantopoulos 2014). These proteins post-transcriptionally regulate iron import, export, and storage genes, ensuring homeostasis (Cassavaugh and Lounsbury 2011).

In granulocytes, heme oxygenase-1 (HO-1) degrades heme into biliverdin, carbon monoxide, and labile Fe^2+^. While HO-1 is induced *via* the nuclear factor erythroid 2-related factor 2 (NRF2) antioxidant pathway and confers cytoprotection by mitigating oxidative stress (Hassannia et al. 2018), excessive HO-1 activity paradoxically elevates labile iron, exacerbating ROS-driven lipid peroxidation and ferroptosis (Chen et al. 2023). This duality manifests in heat stress cases: NRF2/HO-1 activation increases Fe^2+^, depletes GPX4, and amplifies lipid peroxidation, promoting ferroptosis (Zhu et al. 2024). Such conditions underscore HO-1’s context-dependent role in ferroptosis, maintaining equilibrium between cytoprotection and cytotoxicity (Suttner and Dennery 1999). The interplay between iron metabolism, oxidative stress, and cellular defence pathways highlights the complexity of ferroptosis regulation. Understanding these mechanisms provides valuable insights for targeting iron dysregulation in the pathogenesis and treatment of animal diseases. These findings underscore the importance of precisely modulating iron metabolism to prevent or harness ferroptosis in veterinary contexts, offering promising avenues for disease intervention.

### 3.2. Lipid metabolism pathway

Ferroptosis is characterised by the pathological accumulation of intracellular lipid peroxides, which exert cytotoxic effects by destabilising cellular membranes (Dixon et al. 2012; von Krusenstiern et al. 2023) (Figure 2). Lipids, essential components of cellular membranes, are crucial for maintaining normal physiological functions (Harayama and Riezman 2018). However, excessive accumulation of lipid peroxides leads to profound structural and functional disruptions, a hallmark of various diseased tissues (Gaschler and Stockwell 2017). When exposed to enzymatic oxidation or affected by free radicals, polyunsaturated fatty acids (PUFAs) undergo peroxidation, forming lipid peroxides (Mortensen et al. 2023). This mechanism is driven by iron, which catalyses the Fenton reaction to generate ROS that initiate and propagate lipid peroxidation (Dixon et al. 2012; von Krusenstiern et al. 2023). The high susceptibility of PUFAs to peroxidation arises from their bis-allylic hydrogen atoms, which are highly reactive with free radicals (Andreyev et al. 2015). This process compromises membrane integrity, ultimately leading to cell death (Yang and Stockwell 2016). The enzymatic oxidation of PUFAs is a key driver of lipid peroxidation in ferroptosis. Lipoxygenases (LOXs) and cytochrome P450 oxidoreductases catalyse the direct oxidation of PUFAs, producing lipid peroxides (PL-PUFA-OOH), highly reactive molecules that cause membrane damage (Ayala et al. 2014; Kagan et al. 2017). In addition, acyl-CoA synthetase long-chain family member 4 (ACSL4) and lysophosphatidylcholine acyltransferase 3 (LPCAT3) facilitate ferroptosis by incorporating PUFAs into membrane phospholipids, increasing their susceptibility to peroxidation (He et al. 2022). These enzymes highlight the tightly regulated and multifactorial nature of lipid peroxidation in ferroptosis. Understanding the interplay between PUFA metabolism, ROS generation, and membrane vulnerability provides essential insights for targeting ferroptosis in veterinary disease models.

### 3.3. Glutathione (GSH)-GPX4 axis

The GSH-GPX4 axis is the primary defence mechanism against ferroptosis (Li et al. 2022) (Figure 2). GPX4 detoxifies lipid hydroperoxides into non-toxic lipid alcohols, thereby preserving membrane stability and preventing cell death (Yang et al. 2014; Tang et al. 2021; Ma et al. 2022). Depletion of GSH (a cofactor for GPX4) or direct inhibition of GPX4 disrupts this protective axis, resulting in uncontrolled lipid peroxidation and ferroptosis (Sun et al. 2018). This dependence on GPX4 emphasizes the importance of redox homeostasis in preventing ferroptotic cell death (Stockwell et al. 2017). Taken together, dysregulated iron metabolism and lipid peroxidation are hallmarks of ferroptosis, driven by the oxidation of PUFAs in cell membranes through enzymatic and non-enzymatic mechanisms. Enzymes such as LOXs, ACSL4, and LPCAT3 tightly regulate the process, while antioxidant systems like GPX4 protect against oxidative damage. Understanding these mechanisms provides insights into the pathophysiology of ferroptosis and its potential implications in veterinary and comparative medicine (Yang et al. 2014; Stockwell et al. 2017).

## 4. Ferroptosis and infectious diseases

Maintaining iron homeostasis is essential for health. Iron deficiency and/or overload are implicated in various pathologies, including infectious diseases (Ullah and Lang 2023). Infectious diseases pose significant economic challenges in veterinary medicine. For instance, the global dairy industry suffers annual losses exceeding $65 billion due to disease outbreaks (Rasmussen et al. 2024). These losses underscore the urgent need for innovative therapeutic strategies targeting host-pathogen interactions. Therefore, understanding the novel pathogenesis mechanisms can help regulate diagnostic and therapeutic approaches. Pathogens have developed numerous methods to acquire iron from their hosts (Skaar 2010). During infections, the innate immune system increases its iron-withholding defenses, creating a dynamic interplay between host and pathogen as they compete for this critical resource (Cassat and Skaar 2013; Ganz and Nemeth 2015). Central to this process is hepcidin, a key regulator of iron metabolism. Initially identified as an antimicrobial peptide in human urine and blood ultrafiltrate, hepcidin orchestrates the systemic reduction of iron availability during infections, thereby limiting microbial propagation (Park et al. 2001).

**Figure 3.**
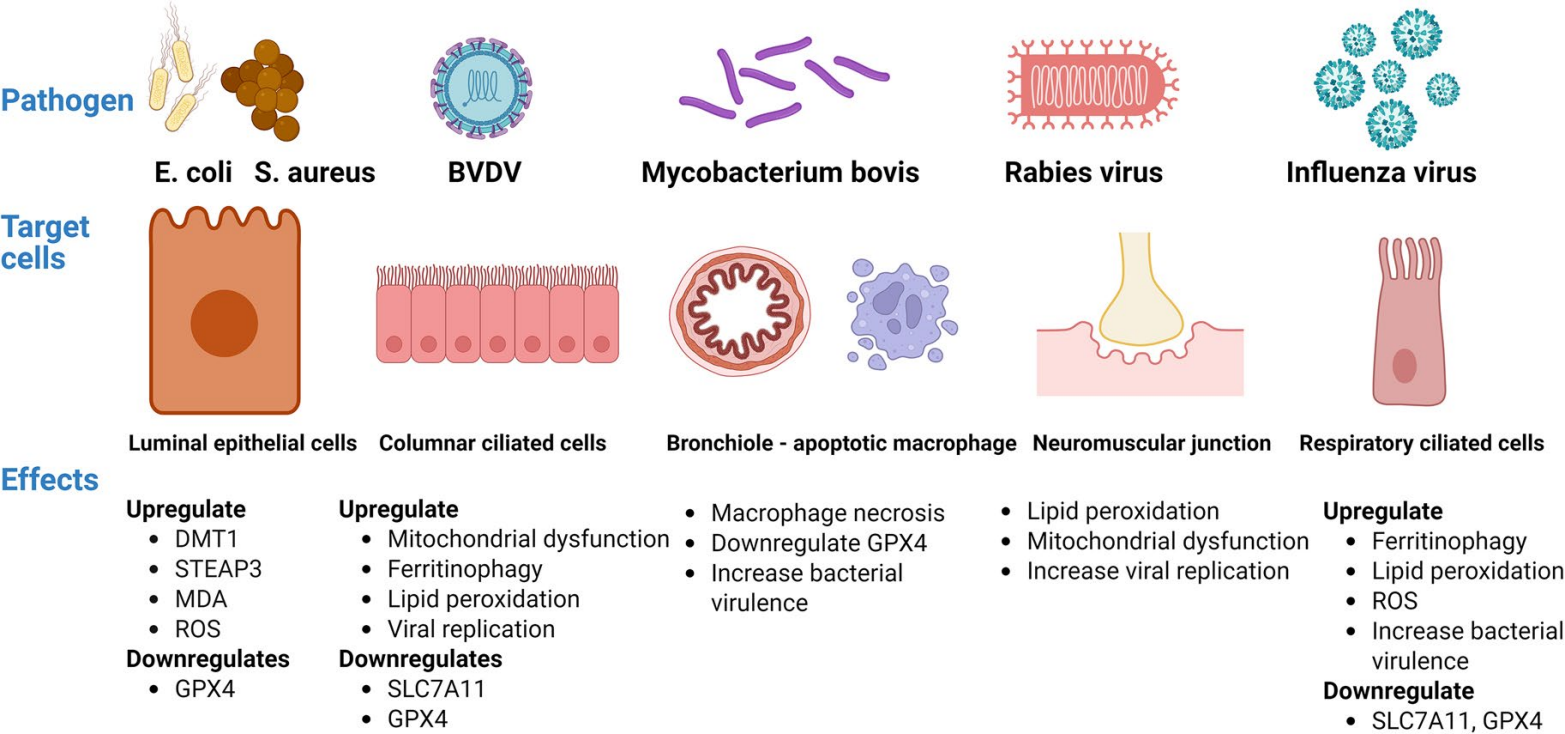
Pathogenic microorganisms hijack ferroptosis pathways, inducing ferroptosis in specific host cells to facilitate the development of infectious diseases. Studies indicate that bacteria and viruses can stimulate animal ferroptosis by affecting lipid peroxidation, interrupting antioxidant systems, and causing mitochondrial dysfunction. DMT1, divalent metal transporter 1; STEAP3, six-transmembrane epithelial antigen of the prostate 3; GPX4, glutathione peroxidase 4; SLC7A11, solute carrier family 7 member 11; MDA, malondialdehyde; ROS, reactive oxygen species. Created with https://www.biorender.com.

Ferroptosis has become a critical pathway influencing infection outcomes (Xiao et al. 2022) (Figure 3). While ROS and iron homeostasis are traditionally linked to host defense, pathogens often use these pathways to promote survival or virulence. Pathogens, however, have developed counterstrategies: some induce ferroptosis to exacerbate tissue damage, while others suppress it to avoid immune detection (Ganz and Nemeth 2015; Silwal et al. 2020). The dual role of ferroptosis underscores the therapeutic potential of its modulators, such as iron chelators and antioxidants. Antioxidants, in particular, exhibit a complex relationship with infection control. For instance, Matsushita et al. (2015) demonstrated that GPX4-deficient T cells, which cannot neutralize lipid peroxides, underwent ferroptosis during infections with acute lymphocytic choriomeningitis virus or Leishmania major. Remarkably, high-dose Vitamin E supplementation restored T cell viability and enhanced pathogen clearance, revealing the delicate equilibrium between oxidative stress and effective immune responses (Matsushita et al. 2015). These findings highlight the importance of regulating iron metabolism and redox balance in infection pathogenesis and support the development of ferroptosis-targeting strategies for infectious disease management in veterinary settings.

### 4.1. Bacterial infections

#### 4.1.1. *Escherichia coli (E. coli), Staphylococcus aureus (S. aureus),* and *Klebsiella pneumoniae*

Bovine mastitis is an inflammatory condition influenced by various factors related to the animal, environment, and pathogens (Plastridge 1958; Cheng and Han 2020; Rifatbegović et al. 2024). It is a primary contributor to economic losses in dairy farming and involves complex interactions between pathogens and the redox systems of the host (Cheng and Han 2020). Accumulation of ROS leads to autophagy-related cell death in bovine mammary epithelial cells (bMECs) through enhanced lipid peroxidation and diminished activity of antioxidant enzymes (Chen et al. 2022). Among the most prevalent environmental pathogens that cause cattle mastitis are *E. coli* (Gao et al. 2017), *S. aureus*, and Klebsiella pneumoniae.

- *E. coli*: Investigations are in progress to assess whether *E. coli* strains obtained from clinical cases of bovine mastitis trigger ferroptosis in bMECs. Zhuang et al. (2024) revealed that *E. coli* activates the Wnt/β-catenin signaling pathway in bMECs, which suppresses GPX4 and promotes ferroptosis (Zhuang et al. 2024). Infected cells showed iron overload due to elevated DMT1 and STEAP3, increased lipid peroxidation indicated by MDA levels, and reduced GSH antioxidant levels (Figure 3). The authors showed that the Wnt pathway inhibitor foscenvivint could alleviate these adverse effects. However, it remains unclear how the efficacy of foscenvivint compares with that of established therapies, including antibiotics and nonsteroidal anti-inflammatory drugs, in mitigating tissue damage.
- *S. aureus* is an extensively studied opportunistic pathogen frequently linked to subclinical mastitis, leading to substantial economic losses due to reduced milk quality and production. The inconsistencies in adhesion and toxin gene variations among bovine *S. aureus* isolates do not reveal clear patterns, complicating association studies related to mastitis and efforts to develop new therapies (Campos et al. 2022). Thus, understanding new pathogenic mechanisms could aid in developing potential treatments. Iron acquisition is essential for *S. aureus* mammary colonization (Cassat and Skaar 2012; Carlson et al. 2020). However, the role of ferroptosis in cell death triggered by *S. aureus* and its relevance in *S. aureus*-induced mastitis are still unclear. Bao et al. (2024) used a mouse model for mastitis along with MECs, showing that *S. aureus* induces ferroptosis, as indicated by increased levels of MDA and a concentration-dependent decline in the production of the antioxidant, GSH (Bao et al. 2024) (Figure 3). The study showed that using a well-known ferroptosis inhibitor such as ferrostatin-1 (Fer-1) and deferiprone significantly decreased cell death caused by *S. aureus* in MECs. This murine model may not accurately replicate bovine mammary pathophysiology, as species-specific immune responses could influence ferroptosis susceptibility. In addition, proteins differentially expressed and associated with iron regulation and apoptosis were identified in cases of clinical mastitis in dairy cows (Zhang Q et al. 2022). In the same study, HO-1 and FTH1 were noted for their involvement in the ferroptosis pathway and exhibited significantly higher expression levels when compared to the control group. To further investigate the effects of antioxidants on an *in vitro* mastitis model using bMECs, Li et al. (2021) found that curcumin reduced ROS accumulation, inflammatory cytokine expression, and lipopolysaccharide-induced apoptosis. These effects were associated with activating the NRF2-antioxidant response element pathway (Li et al. 2021). These findings suggest that antioxidants could be viable alternatives to standard antimicrobial therapies. In a recent study, Xing et al. (2025) investigated host genetic factors influencing *S. aureus* infections in the bovine mammary gland (Xing et al. 2025). Notably, ferroptosis-related genes (HMOX1, SAT1, SLC11A2, STEAP3, and VDAC2) were consistently upregulated throughout infection stages, with the highest expression observed in clinical mastitis. These genes, linked to ferroptosis regulation, represent promising targets for breeding strategies to enhance resistance to *S. aureus*-induced bovine mastitis (Xing et al. 2025).
- Klebsiella pneumoniae: When Klebsiella pneumoniae infected bMECs, it caused significant oxidative stress. This stress reduced levels of key antioxidant proteins, NRF2, KEAP1, HO-1, and the activity of the antioxidant enzyme superoxide dismutase (SOD), while increasing harmful ROS. Interestingly, the infection also lowered levels of proteins involved in ferroptosis, GPX4, ACSL4, and S100A4 (Liang et al. 2025). In the same study, the infection similarly decreased levels of the antioxidant regulator NRF2 and the ferroptosis-inhibiting protein GPX4 in the mammary tissue of the mouse model. Treating the infected cells or the mice *in vivo*, with extracellular vesicles effectively reduced the oxidative stress and the ferroptotic cell death caused by the *K. pneumoniae* infection (Liang et al. 2025).

Collectively, the involvement of ferroptosis across multiple bacterial pathogens highlights its potential role in the pathogenesis of bovine mastitis (Table 1). Modulating ferroptosis through antioxidants, genetic selection, or signaling pathway inhibitors may represent promising strategies to enhance udder health while reducing antibiotic dependency in the dairy industry.

**Table 1.** Ferroptosis contributes to the pathogenesis of animal infectious diseases.

Pathogen		Model	Pathway	Outcomes	Refs.
Bacterial	Escherichia coli	Mammary epithelial cells (MECs)	Wnt/β-catenin signaling pathway	Increased lipid peroxidation, DMT1, STEAP3, and MDA Inhibited system Xc^−^/GPX4 axis Inhibited FPN1 protein expression	(Zhuang et al. 2024)
	Staphylococcus aureus	MECs and the mouse model	Endoplasmic Reticulum Stress by elevated p-PERK-p-eIF2α-ATF4-CHOP	Increased MDA levels and reduced GSH levels	(Bao et al. 2024)
		Clinical mastitis	HO-1 and FTH1	Identified proteins differentially expressed and associated with iron regulation and apoptosis	(Zhang et al. 2022)
		MECs	NRF2-antioxidant response element pathway	Antioxidant, Curcumin, reduced ROS accumulation, inflammatory cytokine expression, and apoptosis	(Li et al. 2021)
		Clinical mastitis	–	Upregulation of HMOX1 and SAT1	(Xing et al. 2025)
	Mycobacterium tuberculosis	Macrophages	GPX4 antioxidant pathway	Promote macrophage necrosis, facilitating bacterial survival and spread	(Amaral et al. 2019)
	Klebsiella pneumoniae	MECs	–	Reduced levels of key antioxidant proteins, increased ROS, and inhibited GPX4	(Liang et al. 2025)
Viral	Bovine viral diarrhea virus	MDBK cells	NCOA4-dependent ferritin degradation	Mitochondrial impairment, iron accumulation, SLC7A11 and GPX4 downregulation	(Li et al. 2024)
	Rabies virus	HEK293 cells	TFR1	Enhanced lipid peroxidation and mitochondrial dysfunction	(Wang et al. 2023)
		N2a and BSR cells	DGAT1/2	Promote cellular lipid peroxidation, Fe^2+^ concentration, and ROS	(Zhao et al. 2024)
	Avian influenza virus	A549 cells	SQSTM1-NRF2-KEAP1 antioxidant pathway	Downregulation of SLC7A11, GPX4, and an increase in ROS	(Wei et al. 2024)
	Newcastle disease virus	Glioma cells	System Xc^-^, NCOA4 and FTH1	Downregulation of SLC7A11 and SLC3A2, depletion of GSH, and increased lipid peroxidation	(Kan et al. 2021)
	Swine influenza virus	A549 cells	System Xc^-^/GPX4 pathway	GSH depletion and an increase in labile iron	(Cheng et al. 2022)
	Severe fever with thrombocytopenia syndrome virus	Vero cells	p62-KEAP1-NRF2 axis pathway	Inhibition of TRIM21 generate sub-lethal levels of ROS	(Choi et al. 2020)
	Porcine epidemic diarrhea virus	Vero cells	Modulation of the ferroptosis pathway	Reduced PEDV replication	(Li et al. 2023)

#### 4.1.2. Mycobacterium tuberculosis (Mtb)

Infections caused by Mtb in animals, especially in bovines, can trigger ferroptosis. During pathogenic infections, cell death often occurs, either aiding the host in eliminating the pathogen or being utilized by the pathogen to increase its virulence (Dar et al. 2018; Dubey et al. 2021). *Mycobacterium bovis* (*M. bovis*) triggers ferroptosis to promote macrophage necrosis, facilitating bacterial survival and spread (Amaral et al. 2019) (Figure 3) (Table 1). This necrotic pathology is detrimental to the host and compromises infection containment. *M. bovis* disrupts GPX4-dependent antioxidant defenses by leveraging its Mb3523c protein to bind host HSP90, thereby destabilizing cellular redox balance (Wang et al. 2025). In murine models, therapeutic interventions targeting this interaction or employing ferroptosis inhibitors like iron chelators and Fer-1 reduce lung necrosis and bacterial burden. Moreover, the addition of antioxidant supplements such as Vitamin E and selenium improves the efficacy of antibiotics in treating tuberculosis. These antioxidants may restore intracellular redox homeostasis, reduce ferroptosis-mediated tissue injury, and enhance host immune resilience. Such combinatorial approaches could be leveraged to shorten treatment duration and mitigate drug resistance. These findings on the molecular processes and roles of ferroptosis triggered by Mtb indicate possible future therapeutic and diagnostic indicators for bovine tuberculosis.

### 4.2. Viral infections

#### 4.2.1. Bovine viral diarrhea (BVD)

BVD, caused by the bovine viral diarrhea virus (BVDV), is a highly contagious disease responsible for significant economic losses in the global cattle industry (Wang and Pang 2024). The pathogenesis of BVD is characterized by a complex interaction between various viral strains, the immune responses of the host, and the timing of infection, leading to outcomes ranging from transient subclinical infections to severe mucosal disease, reproductive failure, and immunosuppression (Lanyon et al. 2014). Studies demonstrate that BVDV uses host autophagy machinery to enhance its replication in Madin-Darby bovine kidney (MDBK) cells while suppressing innate immune defenses (Zhou et al. 2017). Therefore, modulation of autophagy, a process used by the innate immune system to combat viral infections, has been explored as a strategy to treat or prevent diseases associated with viral infections. In addition, BVDV infection induces apoptosis in lymphocytes *via* activation of effector caspase-3, a mechanism linked to immune cell depletion during infection (Pedrera et al. 2012). However, the role of ferroptosis in BVDV pathogenesis remained unclear until recent findings by Li et al. (2024). Their work revealed that both cytopathic and non-cytopathic BVDV biotypes trigger ferroptosis in MDBK cells, marked by mitochondrial dysfunction, ferritinophagy-driven iron overload, elevated lipid peroxidation, and increased viral replication (Li et al. 2024) (Figure 3). In the same study, BVDV induced NCOA4-dependent ferritin degradation, releasing free iron into the cytoplasm and suppressing the NRF2 antioxidant pathway. This suppression downregulated key ferroptosis regulators, including SLC7A11 and GPX4. In contrast, Fer-1 countered these effects, restoring antioxidant defenses and mitigating viral proliferation. This demonstrates that ferroptosis is not only a byproduct of viral pathogenesis but may be actively manipulated by BVDV to enhance its replication and immune evasion. Moreover, the protective effect of Fer-1 suggests that pharmacological inhibition of ferroptosis could serve as an adjunct strategy in managing BVD, potentially limiting viral spread and tissue damage. Currently, no evidence demonstrates the occurrence of ferroptosis *in vivo* during BVDV infection or that its inhibition alters disease outcomes. Future studies should investigate whether similar ferroptosis mechanisms are involved in other viral infections and assess their *in vivo* relevance. Moreover, the interplay between apoptosis and ferroptosis during BVDV infection warrants further investigation.

#### 4.2.2. Rabies virus (RABV)

RABV, a neurotropic pathogen responsible for rabies in humans and animals, is nearly 100% fatal once clinical symptoms manifest. Despite its severity, the molecular mechanisms driving RABV pathogenesis remain incompletely understood, and no effective therapies exist to halt disease progression (Mahadevan et al. 2016). Viral strategies modulate engagement with host redox balance and iron metabolism. For instance, TRIM21, known for degrading interferon regulatory factor 7 and inhibiting antiviral defenses, exacerbates oxidative stress during RABV infection (Zhang B et al. 2023). Another study, reported by Chi et al. (2023), highlighted an antioxidant inflammatory modulator as a potent anti-RABV (Chi et al. 2023). Bardoxolone methyl, an activator of NRF2, was found to inhibit RABV replication *in vitro*, underscoring the potential of redox-targeted treatments as effective therapeutic options (Chi et al. 2023). In ferroptosis, RABV uses the iron metabolism of the host to enhance its infection and promote ferroptosis. Wang et al. (2023) showed that the viral glycoprotein interacts with TFR1, facilitating clathrin-mediated endocytosis and iron absorption (Wang X et al. 2023) (Figure 3) (Table 1). This iron overload drives lipid peroxidation and mitochondrial dysfunction, which may facilitate viral dissemination. Notably, Ding (2024) suggests modulating ferroptosis could offer dual benefits: disrupting viral replication and mitigating neurotoxicity (Ding 2024). Supporting this, Zhao et al. (2024) identified Z-Ligustilide, a bioactive compound from *Ligusticum chuanxiong Hort*, as a potent ferroptosis inducer that suppresses RABV replication by elevating lipid peroxidation, Fe^2+^ levels, and ROS (Zhao et al. 2024). Furthermore, Z-Ligustilide enhanced ACSL4 expression and ferroptosis induction, suggesting that targeted activation of ferroptotic pathways may represent a novel antiviral strategy against RABV.

#### 4.2.3. Avian influenza virus (H5N1)

In humans, the relationship between iron metabolism and the pathogenesis of influenza A virus infections has been investigated, showing that influenza A virus infections alter iron metabolism in the airways and lungs (Pillar et al. 2025). Although influenza A virus infection is known to induce ferroptosis, the precise mechanisms by which the virus causes this pathway remain incompletely understood (Letafati et al. 2024). The influenza virus leads to various forms of cell death, such as apoptosis, necrosis, and ferroptosis (Downey et al. 2018). The latter is driven by iron dysregulation, ROS accumulation, and lipid peroxidation, processes critical for viral pathogenesis (Letafati et al. 2024) (Figure 3). Notably, Ouyang et al. (2024) demonstrated that the influenza virus enhances replication by inducing ferritinophagy (selective degradation of ferritin), exposing cells to ferroptosis (Ouyang et al. 2024). The study found that hemagglutinin is a central factor in this mechanism, which leads to cellular lipid peroxidation. This can delay mitochondrial antiviral signaling protein-mediated immune responses, further facilitating viral spread.

Over the last century, avian influenza outbreaks have been caused by various H5 subtype viruses across many countries and regions (Paul et al. 2019). Epidemiological studies indicate that human infections are linked to contact with virus-infected poultry or contaminated environments (Charostad et al. 2023). H5N1 manipulates host iron and lipid metabolism while suppressing antioxidant defenses to induce ferroptosis (Kung et al. 2022). H5N1 infection promoted ferroptosis in A549 cells by interfering with the SQSTM1-NRF2-KEAP1 antioxidant pathway, as demonstrated by Wei et al. (2024) (Wei et al. 2024). The virus blocks NRF2, resulting in the downregulation of antioxidant genes, SLC7A11 and GPX4, and an increase in ROS, creating a pro-ferroptotic state (Wei et al. 2024) (Table 1). The study showed that the overexpression of TRIM16 restored NRF2 activity, leading to a reduction in ROS and viral replication. On the other hand, H5N1 upregulated TRIM21, which negatively regulates NRF2, indicating a viral strategy to increase oxidative stress and enhance its pathogenicity. These data suggest that ferroptosis contributes to cellular injury during infection and functions as a viral amplification mechanism, positioning redox imbalance as a pivotal target for therapeutic intervention in H5N1-associated diseases. These findings underscore redox imbalance as a strategic target for antiviral interventions.

#### 4.2.4. Newcastle disease virus (NDV)

NDV is a paramyxovirus that affects large poultry populations and has been studied as a potential oncolytic agent due to its selective replication in tumour cells (Mansour et al. 2011). While NDV traditionally induces caspase-dependent apoptosis or ATM kinase-mediated DNA damage in cancer cells (Ren et al. 2020), recent studies highlight its ability to drive oncolytic ferroptosis in glioma cells. Kan et al. (2021) demonstrated that NDV depletes GSH by downregulating system Xc^-^, SLC7A11, SLC3A2, and activating p53, thereby elevating lipid peroxidation (Kan et al. 2021). NDV also induces ferritinophagy (*via* increased NCOA4 and decreased FTH1), exacerbating iron-driven ROS production (Kan et al. 2021). Silencing p53 or NCOA4 reversed these effects, suggesting dual pathways to optimize NDV’s oncolytic efficacy. These findings indicate that ferroptosis and apoptosis complement NDV-induced tumor suppression. Moreover, targeting ferroptosis-related pathways may enhance the therapeutic benefits of NDV-based virotherapy in treatment-resistant malignancies. This ferroptosis-driven mechanism expands its therapeutic potential in oncology.

#### 4.2.5. Swine influenza virus (SIV)

The influenza viruses that are significant in swine are all type A, including subtypes H1N1, H1N2, and H3N2, predominantly infecting respiratory epithelial cells and causing necrotising bronchitis, bronchiolitis, and interstitial pneumonia (Janke 2014). Viral replication and innate immune-driven inflammation, mediated by leukocyte infiltration and cytokine release, synergistically contribute to tissue damage and cell death (Cui X et al. 2024). It has been shown that ferroptosis plays a critical role in determining infection outcomes. For instance, SIV induces ferroptosis in A549 cells by inhibiting the system Xc^-^/GPX4 antioxidant pathway, leading to GSH depletion and increased labile iron (Cheng et al. 2022). These alterations are linked to increased levels of pro-inflammatory cytokines such as TNF-α and IL-6 and enhanced viral replication. Fer-1 reverses these effects, underscoring ferroptosis as a therapeutic target for mitigating influenza-associated pathology. These findings highlight the multifaceted role of ferroptosis in amplifying inflammation and promoting viral propagation during SIV infection. Thus, ferroptosis inhibitors not only reduce oxidative stress but may also serve as immunomodulatory agents in veterinary antiviral strategies.

#### 4.2.6. Severe fever with thrombocytopenia syndrome virus (SFTSV) and porcine epidemic diarrhea virus (PEDV)

Several viruses employ different mechanisms to influence ferroptosis. SFTSV, a viral disease transmitted by ticks and associated with acute fever, inhibits TRIM21 (Choi et al. 2020) (Table 1). This inhibition raises ROS levels to sub-lethal thresholds, enhancing the metabolism of host cells to support viral replication while avoiding cell death (Choi et al. 2020). Conversely, PEDV, a significant threat to swine production that causes diarrhea and mortality in newborn piglets, is sensitive to ROS. Ferroptosis inducers such as erastin and RSL3 markedly reduced PEDV replication upon entering Vero cells, partly through the modulation of the ferroptosis pathway, suggesting that erastin might be a potential therapeutic agent for PEDV infection. In contrast, antioxidants like deferoxamine and N-acetylcysteine promote viral proliferation (Li Y et al. 2023; Zhang H et al. 2023) (Table 1). These findings highlight the virus-specific impact of ferroptosis modulation, indicating that therapeutic strategies targeting ferroptosis must be tailored to the unique pathogenesis of each virus.

## 5. Ferroptosis and non-infectious diseases

### 5.1. Ferroptosis and mycotoxins

Mycotoxins are harmful metabolites produced by fungi that naturally develop on animal feed when environmental conditions are favourable, especially with high moisture and temperatures (Pier et al. 1980; Yang et al. 2020). These compounds are typically low-molecular-weight, non-antigenic molecules and exhibit remarkable heat stability, allowing them to persist through feed processing (Zain 2011). Acute mycotoxicosis occurs when animals ingest sufficient quantities of mycotoxins, resulting in significant organ-specific damage, such as liver necrosis, kidney dysfunction, or neurological issues, all directly related to the biological targets of the toxins (Zain 2011; Xu et al. 2022). However, mycotoxin concentrations in feedstuffs more commonly fall below acute toxicity thresholds. At subclinical levels, their effects become indirect and systemic: they impair growth in young animals, disrupt immune function, and heighten susceptibility to infectious agents by compromising innate resistance mechanisms (Pier et al. 1980; Yang et al. 2020). These secondary effects are often ignored, as visible symptoms typically arise from secondary infections rather than direct exposure to mycotoxins (Yang et al. 2020; Xu et al. 2022). Susceptibility varies across species and age groups, with younger animals generally exhibiting increased sensitivity to specific mycotoxins compared to mature animals. Iron plays a crucial role in biological interactions, including the virulence of the Aspergillus fumigatus, suggesting that fungal iron homeostasis is a promising target for improvement of antifungal treatment and diagnosis of fungal infections (Misslinger et al. 2021). Deoxynivalenol (DON), a widespread Fusarium mycotoxin, intensifies intestinal ferroptosis in piglets by disrupting iron homeostasis and redox balance (Liu et al. 2023b). Liu et al. (2023) revealed that exposure to DON raised ferroptotic markers, including increased MDA levels and decreased GSH, alongside increased gut permeability (shown by elevated serum lipopolysaccharides). DON increased expression of the iron importer DMT1 while suppressing the iron exporter FPN1 and protective mitochondrial proteins (CISD1, FSP1), leading to iron overload. A compensatory rise in ferritin levels indicated a compensatory response for iron storage to alleviate toxicity. Using the iron chelator deferoxamine significantly lessened ferroptosis in intestinal epithelial cells, suggesting that iron modulation could be a viable therapeutic option. These findings position ferroptosis as a mechanistic link between dietary mycotoxin exposure and gut barrier dysfunction in livestock, offering a new target for nutritional or pharmacological interventions.

### 5.2. Ferroptosis in the male reproductive system

The viability of sperm cells plays a crucial role in determining the success of artificial insemination. Sperm cells depend on the balance between ROS and antioxidant mechanisms (Kujoana et al. 2024). While ROS are natural byproducts of oxidative phosphorylation, a metabolic pathway sperm cells depend on for energy production, their relationship with fertility is complex (Zhu et al. 2019). Notably, sperm showing the highest levels of oxidative phosphorylation are often the most fertile (Gibb et al. 2014). However, this metabolic efficiency also increases lipid peroxidation. Oxidative stress, arising from excess ROS or inadequate antioxidant defense, poses a significant threat to sperm function. GSH, the main antioxidant in sperm cells, plays a key role in counteracting oxidative damage, thereby preserving sperm viability and functionality (Ortega-Ferrusola et al. 2019). The SLC7A11-Xc channel provides cystine that helps synthesize GSH (Hashiguchi et al. 2025). Disruption of this system through erastin-induced inhibition depletes GSH and triggers ferroptosis (Tang et al. 2021), which can cause a considerable quantity of sperm death (Table 2).

**Table 2.** Ferroptosis plays a key role in driving cell/tissue death and injury in various physiological systems.

System	Cell/tissue	Model	Pathway	Outcomes	Ref.
Male reproductive	Sperm cells	High glucose levels	LKB1/AMPK	Reduced expression of SLC7A11 and GPX4, increased ROS and MDA levels, and decreased sperm motility	(Li et al. 2023, Martín-Cano et al. 2024)
	Sertoli cells	Heat stress	CYP2C9-Ras-JNK pathway	Elevated CYP2C9, ROS, and TFR1 levels, decreased GPX4 and ferritin levels, and reduced cell viability	(Yang et al. 2024)
	Seminiferous tubules and testes	Zinc exposure	Mitophagy	Decreased the expression of GPX4, ferritin, and SLC7A1, damage to the seminiferous tubules and fiber deposition	(Li et al. 2023)
	Sheep sperm	Cryopreservation	Cleaved-caspase3 and Transferrin receptor	Ferrostatin-1 (Fer-1) demonstrated improved sperm motility and membrane integrity, reduced ROS, lipid peroxidation, and Fe^2+^ levels	(Hai et al. 2025)
Female reproductive	Oocytes	FAC treatment/toxicity	Iron overload	Mitochondrial dysfunction, elevated ROS Compromised Oocyte quality	(Hu et al. 2021)
	Blastocyst	FSP-1	GSH-independent ferroptosis	Elevated iron accumulation, MDA, and MMP levels Impaired blastocyst formation	(Wang et al. 2024)
	Ovaries/granulosa cells	Aged ovaries	PLPP3 upregulation	Accumulated the lipid hydroperoxides, increased levels of MDA, and iron Decreased expression of SLC7A11, FSP1, FTH1	(Quan et al. 2024)
Digestive system	Adipocytes	Clinical Ketosis	AMPK-NFE2L2 complex	Increased abundance of AMPK and NFE2L2. In bovine adipocytes, treatment with H_2_O_2_: Increased Fe²⁺, total iron, ROS, MDA, and ACSL4	(Xu et al. 2021)
Muscular atrophy	Pigs	Selenium-deficient	–	Inflammation and cell death are associated with alterations in the ECM of the muscles	(Zhang et al. 2022)
	Nutritional muscular dystrophy of chicks	Selenium and Vitamin E deficiency	–	Muscle damage, increased oxidative stress markers, and reduced antioxidant capacity	(Huang et al. 2015)
	Satellite cells (SC)		Deletion of TFR1	Undermined SC functionality and muscle repair	(Ding et al. 2021)
Cardiovascular system	Right ventricular failure	Pulmonary artery banding	Fer-1	Fer-1 combated mitochondrial dysfunction and improved the right heart structure and function	(Kazmirczak et al. 2024)

**Figure 4.**
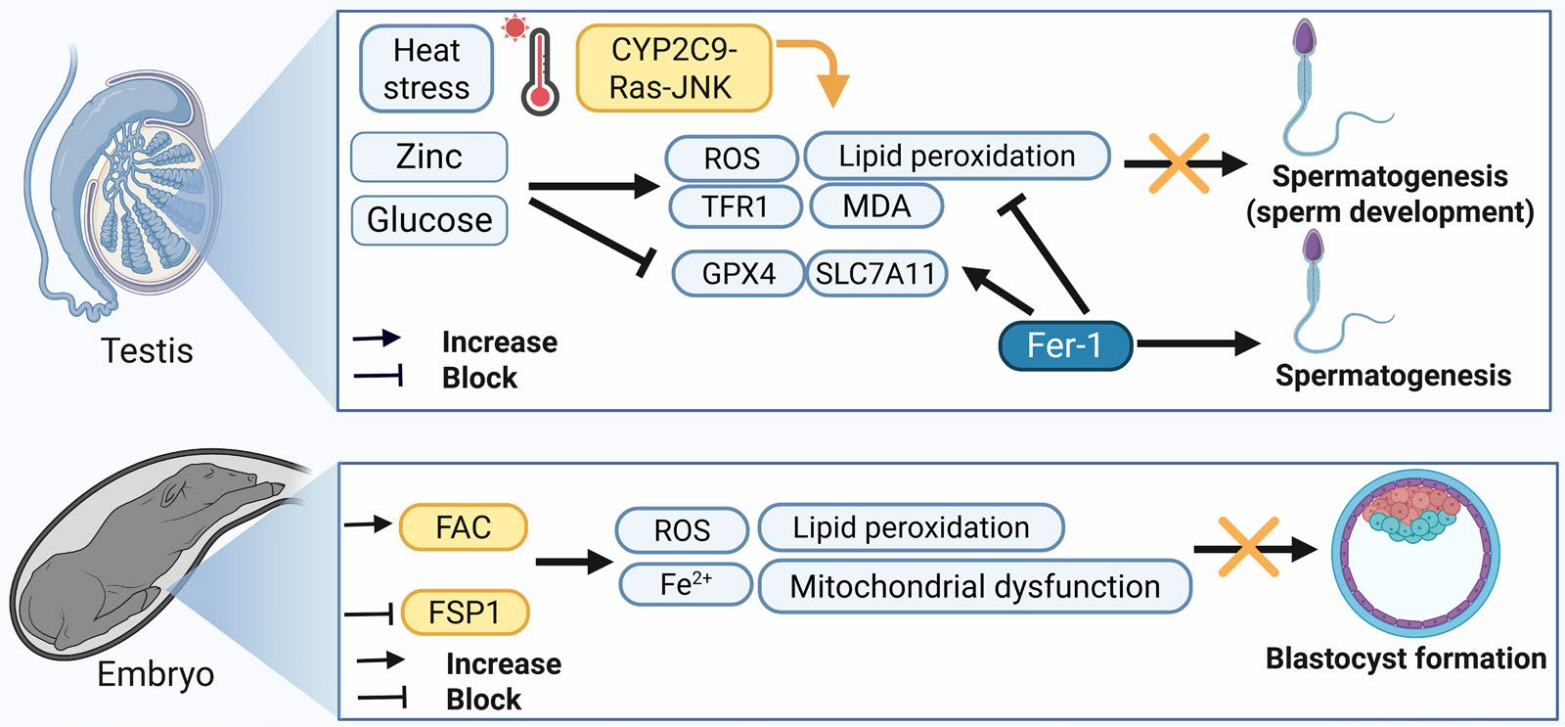
Involvement of ferroptosis in spermatogenesis and embryo maturation. The upper panel shows the effect of ferroptosis triggers, heat stress, and overload of zinc and glucose in normal spermatogenesis. Ferrostatin (Fer-1) can mitigate these adverse effects. The lower panel shows the effect of ferroptosis inducers, ferric ammonium citrate (FAC), and the blocking of ferroptosis suppressor protein-1 (FSP1) in blastocyst formation and impaired embryonic proliferation. TFR1, transferrin receptor-1; GPX4, glutathione peroxidase 4; SLC7A11, solute carrier family 7 member 11; MDA, malondialdehyde; ROS, reactive oxygen species. Created with https://www.biorender.com.

Cryopreservation or refrigeration is commonly used to transport and preserve sperm. However, these methods can expose sperm cells to an osmotic imbalance (Sieme et al. 2008) or harmful cryoprotectants (Sieme et al. 2016). To mitigate these challenges, sperm extenders are regularly used to extend the viability of sperm cells (Bustani and Baiee 2021). Many of these extenders contain glucose concentrations exceeding physiological levels. Recent studies propose that elevated glucose levels can trigger ferroptosis in stallion sperm cells (Martín-Cano et al. 2024) (Figure 4). Several studies found that stallion sperm exposed to high-glucose environments exhibited increased necrosis, ferroptosis markers, and dysregulation of oxidative stress-related enzymes (Martín-Cano et al. 2024). On the other hand, in goat sperm, Li et al. (2023) found that glucose deprivation through low glucose media suppresses ferroptosis, improves rapid linear motility, and increases ATP and mitochondrial membrane potential levels by stimulating oxidative phosphorylation and glycolysis (Li Y et al. 2023). This protective effect is mediated by activation of the LKB1/AMPK pathway, which promotes mitochondrial efficiency and reduces oxidative damage. In contrast, elevated glucose levels in the media and erastin result in decreased expression of LKB1/AMPK, SLC7A11, and GPX4, increased ROS and MDA levels, and significantly impaired sperm motility.

Sertoli cells provide nutritional support and maintain structural integrity during sperm development (Crisóstomo et al. 2018). The testes of mammals, especially Sertoli cells, are particularly susceptible to heat stress, which can cause cellular damage, disrupt functionality, and decrease cell numbers, ultimately leading to inferior sperm quality (Shahat et al. 2020). Research by Yang et al. (2024) revealed that heat stress induced ferroptosis through the cytochrome P450 2C9 (CYP2C9)-Ras-JNK pathway in porcine Sertoli cells (Yang et al. 2024) (Figure 4). This pathway was associated with increased CYP2C9, ROS, TFR1, and epoxyeicosatrienoic acids (EETs), metabolites derived from arachidonic acid. At the same time, GPX4 and ferritin levels declined, contributing to a reduction in cell viability. The adverse effects were counteracted by Fer-1 (Figure 4). Sulfaphenazole, a CYP2C9 inhibitor, lowers EET levels, blocks the Ras-JNK pathway, and prevents ferroptosis, thereby improving cell viability under heat stress. Similarly, Salirasib acts as a Ras inhibitor, enhancing cell viability by obstructing JNK phosphorylation, decreasing ROS levels, and preventing heat stress-induced ferroptosis while not impacting EET levels or CYP2C9 expression. These findings highlight the therapeutic potential of targeting this pathway to protect testicular function during heat stress. Li et al. (2024) demonstrated that fluoride exposure induces male reproductive toxicity in a mouse model and in Leydig cells, primarily through ferroptosis (Li et al. 2024a). Riboflavin mitigated fluoride-induced damage by reducing oxidative stress and inhibiting ferroptosis markers in testicular Leydig cells.

The testes are the primary organs responsible for male reproductive functions and have an endocrine role in regulating male growth and physical characteristics. Zinc is a trace element necessary for reproductive health. However, excessive amounts can lead to adverse effects such as oxidative damage and mitochondrial dysfunction (Marín de Jesús et al. 2024). Elevated seminal zinc correlates with reduced sperm motility, as excessive zinc promotes ROS accumulation, lipid peroxidation, and GPX4 depletion (Li Q et al. 2023) (Figure 4). Studies in young boars revealed that high zinc intake triggers excessive autophagy and mitophagy (selective degradation of mitochondria), leading to iron accumulation *via* ferritinophagy (Li Q et al. 2023). Furthermore, high zinc exposure caused damage to the seminiferous tubules and fiber deposition in a dose-dependent manner in young boars (Li Q et al. 2023). Molecular analyses showed reduced expression of ferroptosis inhibitors, GPX4, ferritin, SLC7A11, and increased markers of ferroptosis, CD71, transferrin, and HMGB1, alongside elevated labile iron pools. Zinc also activated mitophagy regulators, PINK1, Parkin, and autophagy-related proteins, ATG5, LC3-II/LC3-I ratio, indicating that zinc-induced mitophagy drives ferritinophagy and subsequent iron dysregulation. Recently, Hai et al. (2025) found that during sheep sperm cryopreservation, both apoptosis and ferroptosis significantly compromise viability (Hai et al. 2025). Interestingly, they showed that Fer-1 markedly improved post-thaw sperm quality and restored GPX4 levels, demonstrating ferroptosis suppression as a practical approach. This strategy offers considerable potential for safeguarding genetic resources in animal breeding and conservation efforts. Together, these findings highlight the interconnected roles of trace metal homeostasis, autophagy, and ferroptosis in maintaining male reproductive health and underscore the risks of mineral imbalance in animal breeding systems.

### 5.3. Ferroptosis in the female reproductive system

The quality of early embryos is critical for successful implantation and offspring health in mammals, as embryogenesis is a tightly regulated process involving complex molecular mechanisms (Namiki et al. 2018). Ferroptosis has emerged as a key disruptor of embryo viability (Table 2). For example, Ferric ammonium citrate (FAC), a trigger for ferroptosis (Zhang et al. 2020), was shown to impair porcine oocyte maturation in experiments by Hu et al. (2021) (Figure 4). FAC-treated oocytes exhibited mitochondrial dysfunction, elevated ROS, intracellular free iron, and autophagy activation.

These disruptions reduced two-cell and four-cell embryo formation rates, diminished blastocyst development, and compromised oocyte quality. Ferroptosis suppressor protein 1 (FSP1) provides a parallel defense mechanism against ferroptosis by acting as an NADH oxidase (Bersuker et al. 2019; Doll et al. 2019), thereby inhibiting ferroptosis without depending on the GSH pathway. Wang et al. demonstrated that inhibiting FSP1 in porcine embryos triggered GSH-independent ferroptosis, marked by iron accumulation, MDA elevation, mitochondrial membrane potential collapse, and reduced SOD and catalase activity (Wang et al. 2024) (Figure 4).

While GPX4 remained stable, GSH depletion, ATP decline, and oxidative stress impaired embryonic proliferation. This was evidenced by reduced cleavage rates (24–48 h), lower blastocyst formation (144 h), smaller blastocyst diameter, and fewer total cells.

Phospholipid phosphatase 3 (PLPP3) is an enzyme that dephosphorylates phospholipids into lipid substrates, which are oxidized by ROS into hydroperoxides, inducing ferroptosis (Porter 1986). Quan et al. (2024) proposed that PLPP3 regulates ovarian aging in pigs. PLPP3 was significantly upregulated in aged ovaries compared to young ovaries (Quan et al. 2024). PLPP3 upregulation amplified oxidative stress, accumulating lipid hydroperoxides, resulting in increased levels of MDA and iron and decreased expression of SOD1 and CAT enzymes. Furthermore, elevated PLPP3 expression increased autophagosome formation and mitochondrial damage, exacerbating ferroptotic cell death in ovarian tissues. PLPP3 upregulation skewed the antioxidant-oxidative stress balance and induced autophagy and ferroptosis.

### 5.4. Ferroptosis and ketosis in dairy cows

Ketosis is a metabolic disorder in dairy cows characterized by negative energy balance (NEB) following parturition and during peak lactation (Zhang et al. 2016). During NEB, lipolysis mobilizes body fat reserves, releasing free fatty acids (FFAs) primarily oxidized in the liver (de Vries and Veerkamp 2000). When circulating FFAs exceed the hepatic oxidation capacity, they accumulate in hepatocytes, inducing metabolic stress (Schulz et al. 2015). Hypoxia-inducible factor-1α (HIF-1α), a transcriptional regulator of cellular hypoxia responses (Semenza 2012), also modulates oxidative stress *via* mitochondrial adaptation and gene regulation (Li et al. 2019). Recent studies highlight its role in ferroptosis inhibition through the HIF-1α/SLC7A11 pathway, which enhances GSH synthesis (Yuan et al. 2022). Adipose tissue from ketotic cows exhibits elevated HIF-1α protein levels alongside markers of ferroptosis and oxidative stress. *In vitro* NEB simulations further demonstrated that HIF-1α upregulation protects adipocytes from ferroptotic damage and oxidative stress. The NFE2L2-KEAP1 complex, a molecular sensor in bovine adipose tissue (Xu et al. 2021), activates antioxidant defenses under oxidative stress by dissociating to trigger the transcription of genes like SOD1, HMOX1, and CAT (Ghareghomi et al. 2023). Adipocytes from ketotic cows showed increased Fe^2+^, total iron, ROS, MDA, and ACSL4. These changes are consistent with ferroptosis activation, highlighting the oxidative imbalance during ketosis (Table 2). Notably, HIF-1α overexpression reversed erastin-induced ferroptosis by restoring SLC7A11 expression, suppressing ACSL4, and reducing ROS, MDA, and iron accumulation. However, clarification is needed regarding the specific effects of HIF-1α and whether the HIF-1α/ferroptosis dynamics occur in hepatocytes. These findings suggest that enhancing the HIF-1α/SLC7A11 axis may represent a promising therapeutic strategy to mitigate ferroptosis-associated adipose dysfunction in ketotic cows. Recently, Wang et al. (2025) investigated similar pathways in metabolic disease, focusing on liver HIF signaling in murine models of diabetes (Wang et al. 2025), which contrasts sharply with findings in bovine adipose tissue. In addition, Wang et al. (2025) likely used dietary or metabolic models (e.g. high-fat or methionine-choline-deficient diets) to induce ferroptosis, offering greater physiological relevance than erastin-based approaches.

### 5.5. Ferroptosis and muscular disorders in animals and chicks

It is well known that a deficiency in dietary selenium leads to nutritional muscular dystrophy (NMD) (Hill et al. 2001), but the exact reasons are not clear. Research on pigs and chicks has provided insights into this issue (Huang et al. 2015; Zhang K et al. 2022). When pigs were fed a severely selenium-deficient diet for 16 weeks, their muscle selenium levels collapsed by 96%, reducing GPX and thioredoxin reductase and activity. It also markedly lowered the expression of numerous selenoprotein genes (Table 2). A deeper analysis using multi-omics approaches revealed shifts in genes, proteins, metabolites, and lipids (Zhang K et al. 2022). This deficiency in selenium affects muscle cells, alters their energy and fat metabolism, increasing glycolysis while decreasing phospholipid synthesis. The resultant oxidative stress led to inflammation and cell death, associated with alterations in the ECM of the muscle, suppression of survival pathways, PI3K/AKT, and activation of harmful pathways like NF-κB. Huang et al. showed that feeding chicks a diet lacking both selenium and Vitamin E caused severe NMD and high death rates (Huang et al. 2015). Selenium-deficient chicks showed apparent muscle damage by week 3, alongside increased oxidative stress markers and reduced antioxidant capacity. Crucially, selenium deficiency knocked down key selenoproteins in the muscle before symptoms appeared. This loss seemed to release damaging pathways, leading to the oxidative damage and cell death characteristic of NMD. Essentially, these selenoproteins act as vital guardians against peroxide damage and redox imbalance in muscle.

Beyond nutritional deficiencies, muscle atrophy poses a significant health issue, significantly affecting mobility and life quality, with no effective treatments available. Recent research points to ferroptosis as a critical mechanism (Wang et al. 2022). Satellite cells (SCs) are essential for muscle repair (Kaczmarek et al. 2021). Their reduction is directly correlated to muscle deterioration. TFR1 is vital for muscle health; deleting it in muscle tissue results in significant dysfunction (Ding et al. 2021). Importantly, TFR1 levels drop significantly in the muscles and SCs of aged mice compared to young ones (Ding et al. 2021). Research that involved the deletion of TFR1 specifically in mouse SCs showed destructive effects; SC numbers irreversibly dropped, and the remaining cells could not properly mature. This failure was linked to ferroptosis (Ding et al. 2021). This cell death, in turn, further undermines SC functionality and muscle repair. While Fer-1 was ineffective in rescuing the TFR1-deleted SCs, directly reintroducing the TFR1 gene into the muscle reduced iron accumulation and enhanced regeneration (Ding et al. 2021). In aged muscle, TFR1 declines while another iron importer (SLC39A14) increases, leading to labile iron accumulation. Here, Fer-1 was effective, significantly improving the running ability of aged mice. This strongly suggests ferroptosis driven by altered iron handling is a fundamental process driving muscle degeneration in aging.

### 5.6. Ferroptosis and cardiovascular diseases

Right ventricular failure (RVF) is the primary driver of mortality in pulmonary arterial hypertension (PAH), a finding consistent across veterinary and human medicine (Prisco et al. 2020). Rodent and human studies implicate ferroptosis in PAH-mediated RVF, which is driven by mitochondrial dysfunction and dysregulated fatty acid oxidation (Stockwell 2022) (Table 2). Kazmirczak et al. tested this mechanism in a porcine PAH model induced by pulmonary artery banding (PAB), demonstrating that Fer-1 improved right heart structure and function (Kazmirczak et al. 2024). Key outcomes included an 11% increase in right ventricular ejection fraction (RVEF), reduced right ventricle and atrium pathological dilation, and preserved mitochondrial cristae integrity. Fer-1 partially restored the expression of oxidative phosphorylation proteins and mitigated PAB-induced disruptions in fatty acid metabolism, underscoring ferroptosis as a therapeutic target for RVF. These findings emphasize the pathological relevance of ferroptosis in cardiovascular remodeling and suggest that targeting lipid peroxidation pathways may offer cardioprotective benefits in PAH-associated RVF.

## 6. Small-molecule inhibitors and nutrition and food additives targeting ferroptosis

Current ferroptosis therapies-small-molecule inhibitors Fer-1, iron chelators (deferoxamine), and genetic modifications-exhibit rapid but transient efficacy in acute models, demanding repeated dosing (Wang et al. 2023; Zhang et al. 2024). Applying their therapeutic potential from *in vitro* and murine studies to everyday veterinary practice faces many challenges. Substantial feasibility barriers further hinder clinical translation. Major obstacles include the poor pharmacokinetic properties of synthetic inhibitors, such as low solubility and metabolic stability (Skouta et al. 2014; Hofmans et al. 2016), which complicates formulation and dosing for veterinary species. For instance, Fer-1 is hampered by poor bioavailability, metabolic instability, and a short plasma half-life (Miotto et al. 2020). The safety profile of most specific ferroptosis inhibitors remains unknown, mainly in veterinary species. While the side effects of chronic iron chelation are serious and well-documented (Mobarra et al. 2016), the long-term toxicity, off-target effects, and safety of novel inhibitors in pregnant animals have not been researched. Iron chelators alleviate overload but disrupt physiological iron balance (Scarpellini et al. 2023). A potential risk is that inhibiting ferroptosis could worsen infectious disease outcomes by allowing infected cells to survive (Ganz and Nemeth 2015; Silwal et al. 2020). Therefore, regulating ferroptosis *via* anti-ferroptotic nutrients in feed is a promising approach, especially in the livestock and poultry industry, where individual treatment of sick animals is impractical. Plant extracts, amino acids, selenium, and Vitamin E are among the nutrients and food additives that target ferroptosis.

*Plant extracts:* Research increasingly points to ferroptosis as a significant factor in animal intestinal damage (Xiao et al. 2025). Finding ways to control this process in gut cells using nutrients or natural compounds could be key to preventing or healing such injuries. Interestingly, several nutrients and plant-based antioxidants promise to regulate ferroptosis (Wu et al. 2024). For instance, polyphenols from the holly plant (Ilex latifolia Thunb.) protected weaned pigs by blocking ferroptosis triggered by the toxin diquat, reducing damage to their small intestine structure and function (He et al. 2020). Hesperidin, a major flavonoid in citrus fruits, could alleviate mitochondrial dysfunction, oxidative stress, and intestinal injury in Deoxynivalenol-challenged piglets (Li et al. 2024b).

*Amino acids:* Glycine, a building block for the antioxidant GSH (McCarty et al. 2018). Xu et al. (2022) investigated whether glycine could lessen the intestinal damage caused by diquat in weanling piglets and the relationship between ferroptosis and diquat-induced intestinal epithelial cell death (Xu et al. 2022). The results showed that dietary glycine reduced intestinal oxidative stress induced by diquat in weanling piglets. Furthermore, with increasing anti-oxidative capacity, dietary glycine could restrain intestinal epithelial cell ferroptosis triggered by diquat. The amino acid glycine is essential due to its antioxidant properties and was reported to play an important role in oocyte maturation and development by regulating ROS-induced lipid metabolism (Gao et al. 2023). Methionine, an essential amino acid used widely in livestock feed, alleviates heat stress-induced ferroptosis in bMECs (MAC-T cell line) (Xu et al. 2023). Methionine enhances GSH synthesis, activating the NRF2 pathway to restore mitochondrial function, reduce ROS, and lower labile iron pools, lipid ROS, and MDA levels. It also upregulates antioxidant enzymes and ferroptosis-related proteins, improving iron storage capacity. GSH itself, vital for maintaining the antioxidant balance of the cell, also combats intestinal damage. In piglets exposed to the toxin paraquat, GSH reduced oxidative stress and damage by increasing GPX4 levels in the small intestine (Xiang et al. 2022). Another study uncovered an interesting mechanism: when piglets lack the amino acid cysteine, dietary GSH can be broken down to supply it (Jiao et al. 2022). Since cysteine is essential for producing GPX4, this process ultimately helps suppress intestinal ferroptosis.

*Selenium and Vitamin E:* Selenium is the primary nutrient that controls ferroptosis, as it is used by cells to produce GPX4, which shields cells from lipid peroxidation and ferroptosis (Conrad and Proneth 2020). In a study on cadmium-induced nephrotoxicity in sheep, cadmium induced ferroptosis by causing iron overload, up-regulating PTGS2, NCOA4, TFR1, and LC3B mRNA levels and PTGS2 and LC3B-II/LC3B-I protein levels, reducing SLC7A11 and FTH1 mRNA and protein levels (Wang L et al. 2023). However, Selenium supplementation increased the expression level of GPX4 and reversed ferroptosis. Dong et al. showed that selenomethionine antagonizes oxidative stress and ferroptosis induced by Decabromodiphenyl ether (BDE209) by activating the NRF2/GPX4 pathway (Dong et al. 2024).

Vitamin E shows promising results in preventing and alleviating ferroptotic effects (Zhang et al. 2024). Dietary supplementation of Vitamin E has a promising effect in blocking ferroptosis, although this has not been reported in veterinary medicine. These findings suggest that nutritional strategies targeting ferroptosis could serve as practical, preventive interventions to enhance animal health and production efficiency.

## 7. Species-specific variations in ferroptosis regulation

Ferroptosis mechanisms, including those in animals, display both conserved characteristics and unique adaptations across different species (Conrad et al. 2018). For example:

- Mammals such as livestock and companion animals heavily depend on GPX4 to detoxify lipid peroxides. However, species variations in selenium metabolism can affect the efficacy of GPX4 (Liu et al. 2022). Ruminants have enhanced systemic iron recycling due to high splenic ferritin storage (Narozhnykh et al. 2024), which may increase susceptibility to ferroptosis during inflammatory conditions such as mastitis. Key regulators such as NRF2 and SLC7A11 demonstrate tissue-specific expression patterns. For example, porcine hepatic cells show strong NRF2-mediated antioxidant responses and greater heat tolerance (Chen et al. 2017).
- Avian species exhibit unique regulation of ferroptosis due to their evolutionary adaptations. Poultry show reduced dependency on GPX4 but possess enhanced alternative antioxidant defenses, such as GST-mediated glutathione recycling (Murcia and Diaz 2021). This adaptation may help mitigate lipid peroxidation during periods of high metabolic stress, like heat exposure (Aryal et al. 2025). Their high metabolic rate and oxygen consumption increase ROS formation. However, constant activation of NRF2 antioxidant pathways helps mitigate oxidative stress (Castiglione et al. 2020).
- In aquaculture species, susceptibility to ferroptosis is influenced by environmental stressors (Xia et al. 2021). Acute waterborne cadmium exposure induces hepatic ferroptosis, potentially via the NRF2/KEAP1 signaling pathway (Chen et al. 2024). Differences in lipid composition, including higher PUFA levels in fish membranes, may further increase sensitivity to lipid peroxidation.

## 8. Novel insights and knowledge gaps

A key new insight in the current review is how species-specific pathogens purposely develop this pathway. They actively manipulate regulators such as SLC7A11 and GPX4 to induce cell death and enhance their virulence, moving beyond viewing ferroptosis as a passive consequence. Furthermore, the review offers a critical comparative perspective for understanding ferroptosis, especially highlighting the significant interspecies differences in susceptibility driven by nutritional factors. The threshold for triggering ferroptosis through dietary deficiencies, such as selenium, varies widely and is affected by metabolic rate and physiological needs; this is particularly clear in fast-growing species like broiler chickens and weanling pigs. These findings highlight that nutritional strategies to prevent ferroptosis cannot be one-size-fits-all and must be carefully tailored for each species. The review also offers valuable naturally occurring models that advance both veterinary medicine and broader biomedical research into redox biology and cell death.

Despite this growing body of evidence, significant gaps in our understanding remain. There are considerable knowledge gaps regarding less-studied animals. Therefore, future research should focus on elucidating the specific molecular mechanisms of ferroptosis across different animal species, as interspecies variations may influence its role in disease progression. For instance:

- In poultry, while lipid peroxidation biomarkers suggest that ferroptosis may play a role in poultry diseases, mechanistic studies lack confirmation.
- Biomarker standardization across species, such as lipid peroxidation markers in biofluids such as plasma and synovial fluid, is needed for the early identification and monitoring of disease development. Such biomarkers could also facilitate the evaluation of treatment efficacy and disease prognosis.
- Mapping ferroptosis regulators across livestock, companion animals, and aquaculture species is essential.
- In aquaculture, research on the role of ferroptosis in viral infections is lacking, and the interactions between dietary iron and antioxidants have yet to be quantified.
- Using nutrients to prevent and treat various animal diseases and improve production is essential. These targeted dietary interventions may provide cost-effective, scalable solutions to mitigate ferroptosis-associated pathologies, particularly in livestock and poultry industries.

## 9. Conclusions and perspectives

Although ferroptosis has attracted increasing attention in human medical research, its investigation within veterinary medicine remains relatively limited. This gap may be attributed to several factors, including a general lack of funding relative to human health studies, clinical priorities in veterinary medicine that focus on disease treatment and prevention over fundamental mechanistic research, a focus on well-established diseases and therapies, and limited interdisciplinary collaboration. Nevertheless, ferroptosis has been identified as a contributing factor in major veterinary diseases, including infectious diseases, reproductive disorders, and organ injuries.

Consequently, there is a growing need to investigate ferroptosis in species-specific contexts. One limitation of the current review is that the field depends on the fundamental insights from *in vitro* and mouse models, a necessary starting point. However, this dependence reflects the early stage of veterinary ferroptosis research, where species-specific mechanistic studies remain limited. Major differences between species in iron metabolism, immune response, and drug pharmacokinetics indicate that significant findings from mouse models require thorough validation in livestock and companion animals. Integrating this research provides a new framework for understanding complex disease mechanisms. Recent data highlight ferroptosis as a promising therapeutic target and a crucial pathway in disease progression. Therefore, ongoing exploration and collaboration between researchers and veterinarians are essential to deepen our understanding of animal disease pathogenesis and to advance precision health strategies in veterinary care.

## Data Availability

The authors confirm that the data supporting the findings of this study are available within the article and/or its supplementary materials.
